# Seropositive Neuromyelitis Optica in a Case of Undiagnosed Ankylosing Spondylitis: A Neuro-Rheumatological Conundrum

**DOI:** 10.5339/qmj.2022.29

**Published:** 2022-07-07

**Authors:** Ritwik Ghosh MD, Devlina Roy, Moisés León-Ruiz, Shambaditya Das, Souvik Dubey, Julián Benito-León

**Affiliations:** ^1^Department of General Medicine, Burdwan Medical College & Hospital, Burdwan, West Bengal, India E-mail: jbenitol67@gmail.com; ^2^Department of Neurology, Calcutta National Medical College & Hospital, Kolkata, West Bengal India; ^3^Section of Clinical Neurophysiology, Department of Neurology, University Hospital “La Paz,” Madrid, Spain; ^4^Department of Neuromedicine, Bangur Institute of Neurosciences, Institute of Post Graduate Medical Education and Research & SSKM Hospital, Kolkata, West Bengal, India; ^5^Department of Neurology, University Hospital “12 de Octubre,” Madrid, Spain; ^6^Centro de Investigación Biomédica en Red sobre Enfermedades Neurodegenerativas (CIBERNED), Madrid, Spain; ^7^Department of Medicine, Complutense University, Madrid, Spain

## Abstract

Neuromyelitis optica spectrum disorder (NMOSD) is an autoimmune astrocytopathy against foot processes of aquaporin-4 (AQP4) water channels. Patients with NMOSD tend to have other coexisting autoimmune/connective tissue diseases. However, AQP-4-antibody-positive NMOSD coexisting with ankylosing spondylitis (AS) is rare. AS is an immune-mediated disorder, a subset of axial spondyloarthropathies, which commonly manifests as chronic inflammatory back pain in young people, and it has a strong association with HLA-B27. In this study, a 35-year-old Indian man with an undiagnosed progressive axial spondyloarthropathy (i.e., AS) is reported presenting with acute-onset longitudinally extensive transverse myelitis, a clinical subset of NMOSD.

Neuromyelitis optica spectrum disorder (NMOSD), a primary demyelinating disorder of the central nervous system (CNS), is an autoimmune astrocytopathy against foot processes of aquaporin-4 (AQP4) water channels, which manifests with optic neuritis, longitudinally extensive transverse myelitis (LETM), area-postrema syndrome, brainstem syndrome diencephalic syndrome, and cerebral syndrome.^1–4^

Ankylosing spondylitis (AS) is an immune-mediated disorder, a subset of axial spondyloarthropathies, which commonly manifests as chronic inflammatory back pain in young people, and it has a strong association with HLA-B27.^5,6^ AS characteristically targets the axial skeleton, peripheral joints, entheses (connective tissues between tendons/ligaments and bones), and gut.^5,6^

Patients with NMOSD tend to have other coexisting autoimmune/connective tissue diseases.^7^ For example, cases with NMOSD and multiple sclerosis, which are other autoimmune primary demyelinating disorders of the CNS, have been reported.^8,9^ However, concurrent existence of AS and NMOSD in the same patient even over years of disease course is rare.^10,11^ In addition, studies describing neurological manifestations of AS are limited,^12^ and they focus on joint inflammation and long-standing bony pathology (ankylosis) related to compressive myelopathy, myelo-radiculopathy, and cauda equina syndromes.^12,13^

The authors present a case of a young Indian man with an undiagnosed progressive AS (misdiagnosed and mismanaged by an indigenous medical practitioner) presenting with acute-onset LETM variant of AQP4-positive NMOSD.

A 35-year-old healthy, non-comorbid man from rural India came to the outpatient department with complaints of persistent tingling, numbness, and weakness of both lower limbs (right more than left) for 10 days. The clinical picture showed acute-onset urinary retention, which was relieved by urinary catheterization. An indigenous medical practitioner had prescribed drugs to treat a urinary tract infection. His weakness gradually progressed over the following week, causing him to become bedridden. During the removal of the catheter, he felt urgency, increased frequency of micturition, and overt urinary incontinence. He gave no history suggestive of any girdle-like sensations, root/radicular/tract pain, vertebral pain, trauma, recent vaccination, and diarrheal or febrile illness.

For the last 8 months, he had a complaint of an insidious-onset, persistent, bilateral, dull aching pain in the gluteal region accompanied by low-back pain and morning stiffness up to 1 h, which markedly improved with activity and reoccurred following long periods of inactivity. He sometimes had to rise in the middle of the night because of excruciating pain, which could be relieved after moving around the room and corridors for half an hour. He was taking over-the-counter diclofenac tablets for pain relief prescribed by some indigenous medical practitioners who told him that it was due to overwork in agricultural fields, that is, mechanical back pain. He also had a normal X-ray of the lumbosacral spine.

He had no addiction liabilities, and none of the family members had ever suffered from a similar kind of illness. He had never consulted any trained medical practitioner, as his previous back-pain-related symptoms responded well to the tablets prescribed by the indigenous medical practitioner(s).

During examination, he was found to have recent-onset, asymmetric spastic paraparesis (right more than left) with upper motor neuron-type urinary bladder symptoms. Cognitive assessment (assessed by the Montreal cognitive assessment test) was normal, and posterior column sensations were preserved. Sensory system examination revealed no definite sensory level. Except for the paretic lower limbs, cerebellar functions were normal in other regions. Neuro-ophthalmological examinations were also normal, and no signs of meningeal irritation were observed.

The history and course of the disease and clinical examinations were analyzed. Selective tractopathy (early and predominant motor and autonomic tract affection) was suggested for an intramedullary demyelinating pathology affecting the anterior central cord. This case was initially classified as acute-onset non-compressive myelopathy at the lower cervical/upper dorsal region level in a patient with a pre-existing axial spondyloarthropathy.

Complete blood cell count; liver, kidney, and thyroid function tests; and plasma glucose and electrolytes were normal, except for an increased erythrocyte sedimentation rate (66 mm in the first hour). Magnetic resonance imaging (MRI) of the spinal cord revealed a demyelinating LETM from C5 to D4 level (Figure 1). Meanwhile, an MRI of the sacroiliac joints revealed bilateral sacroiliitis. Brain and orbital MRIs were devoid of any lesions. Anti-aquaporin 4 (AQP-4) antibodies were tested by cell-based assay in serum and cerebrospinal fluid (CSF), and both were positive. CSF further revealed lymphocytic pleocytosis and increased intrathecal protein production. Visually evoked potential recordings were also normal. In addition, anti-myelin oligodendrocyte glycoprotein antibodies were negative. Anti-nuclear antibody (ANA), ANA-profile, autoimmune vasculitis profile (c-ANCA, p-ANCA), neurovirus panel (i.e., polymerase chain reaction for adenovirus, Epstein–Barr virus, herpes simplex viruses 1 and 2, human herpesviruses 6 and 7, cytomegalovirus, enteroviruses, varicella-zoster virus, Japanese encephalitis, and dengue virus), CSF-polymerase chain reaction for *Mycobacterium tuberculosis*, angiotensin-converting enzyme, anti-phospholipid, and anti-thyroid antibodies were negative. Anti-CCP-antibody and rheumatoid factor were also negative, including creatine phosphokinase level and serum vitamin B12. Moreover, serologies for hepatitis B, C, human immunodeficiency virus, and scrub typhus were negative. However, HLA-B27 assay was positive. The final diagnosis was AQP4-positive NMOSD associated with AS. He was placed on pulse intravenous methylprednisolone (1 g/day for 5 days). Consequently, his lower limb power improved remarkably. Cyclical rituximab therapy was initiated to prevent relapses. At 3-month follow-up, he had no residual neurological deficit except for persistence of paresthesias. Neuroimaging and visually evoked potential studies revealed no active or new lesions. After 6 months of therapy, a subjective and objective improvement was observed in disease severity based on the Ankylosing Spondylitis Disease Activity Score.

Our patient satisfied the new Assessment of SpondyloArthritis International Society diagnostic/classification criteria for AS and the Wingerchuk criteria for NMOSD,^4,14^ an association that has been rarely reported.^10,11^

Amid the extra-articular complications of long-standing AS, neurological manifestations are considered infrequent.^15^ However, subclinical neurological complications may be frequent in AS.^12^ Common neurological manifestations result from bony (vertebral) ankylosis, subluxation of joints, ossification of anterior and posterior longitudinal ligaments, secondary spinal canal stenosis, bony (vertebral) fractures, and subsequent compressions over nerve radicles/roots/cauda equina, and inflammation-related (entrapment) peripheral neuropathies.^12,16,17^ Acute transverse myelitis can occur as a subset of several primary demyelinating disorders of the CNS (i.e., multiple sclerosis, NMOSD, myelin oligodendrocyte glycoprotein antibody disease, and acute disseminated encephalomyelitis) and various systemic autoimmune connective tissue disorders (i.e., systemic lupus erythematosus, mixed connective tissue disease, Sjögren syndrome, inflammatory bowel disease, and neurosarcoidosis).^18^ Acute transverse myelitis (short or long segment) is an infrequent extra-articular complication of AS.^18^ It has been reported to evolve either as a distinct neurological complication of AS, or it may develop secondary to TNF-alpha-inhibitor therapy for the treatment of AS.^18,19^

AS is a heritable inflammatory spondyloarthropathy that primarily affects the axial skeleton, which is mediated by T-cells; B-cells only play a minor role.^5^ On the contrary, the key for the pathogenesis of NMOSD is the production of autoantibodies against AQP-4 channels expressed on astrocytes, leading to complement-mediated damage, with ensuing demyelination. Myelitis usually shows high signal intensity on the tbl2‐weighted image and contrast enhancement in the spinal cord.^1–4^

Despite the difference in molecular mechanisms, the diagnosis of these diseases in the same individual may not be coincidental. Recent evidence has shown T-cell-mediated inflammatory responses in cases of NMOSD.^20^ In particular, Th17 and Th2-related cytokines are elevated in the CSF of NMO patients.^20^ Environmental factors such as *Escherichia coli* have also been proven to aggravate autoimmunity in AS and NMOSD (however, body fluid cultures for *Escherichia coli*, performed in our patient, showed similar association, and they were found negative two times).^21,22^ Although large-scale epidemiological studies investigating the underlying pathogenesis related to these diseases are lacking, studies have demonstrated an increased incidence of optic neuritis among patients with AS.^23^

Systemic sclerosis and mixed and undifferentiated connective tissue diseases were excluded after expert opinions (from two board-certified rheumatologists and two dermatologists) because of the lack of suggestive clinical findings (e.g., absence of skin thickening, salt-and-pepper appearance, nail changes, Mauskopf facies, sclerodactyly, calcinosis cutis, Raynaud’s phenomenon, other cutaneous manifestations, pulmonary arterial hypertension/interstitial lung disease, dysphagia, muscular pain/weakness renal impairments, absence of ANA, anti-centromere antibodies, anti-Scl-70, PM-Scl antibodies, anti-ds DNA, PCNA, CENP-B, anti-nucleosomes, anti-Smith, anti-U1-RNP, anti-Jo1, anti-Mi2, anti-Ro52, anti-La antibodies, and normal C3 and C4 complement levels) (The European League Against Rheumatism and the American College of Rheumatology classification criteria 2019).^24^

Finally, our patient was treated with intravenous steroids followed by rituximab infusions, a monoclonal anti-CD20 antibody directed against B-cells. In particular, this patient clinically and radiologically responded to immunomodulatory drugs, which might support a possible common pathogenic basis of the two processes. TNF-alpha inhibitors are commonly used as novel therapeutics in AS; however, they can potentially result in serious complications, that is, secondary demyelinating disorders.^25^ However, such inhibitors in this patient were not used. When used in cases of AS, they show satisfactory results.^25,26^ Therefore, it was decided to treat him with rituximab only without adding any second immunomodulatory. Other possible therapeutic options include cyclophosphamide and mycophenolate mofetil, but they were not used because of their low efficacy–safety balance. Moreover, plasmapheresis was not available in our specific setting, despite solid evidence that early treatment with therapeutic strategy (5–7 courses) provides good long-term outcomes in patients with NMOSD.^27^

Therefore, when dealing with a case of acute non-compressive myelopathy, history and clinical examination are important to determine the potential underlying etiology and identify an undermined systemic disorder with apparently unrelated non-specific features. Connective tissue disorders should always be considered as a differential diagnosis and be ruled out in all cases of either seropositive or seronegative NMOSD. A diagnosis of AS should be considered in relevant circumstances when dealing with a case of isolated seronegative LETM. Moreover, early diagnosis and treatment of AS are quintessential to prevent lifelong distressing disabilities. However, whether patients with AS have any extra predilection to develop NMOSD throughout their life requires further studies.

## Figures and Tables

**Figure 1.**
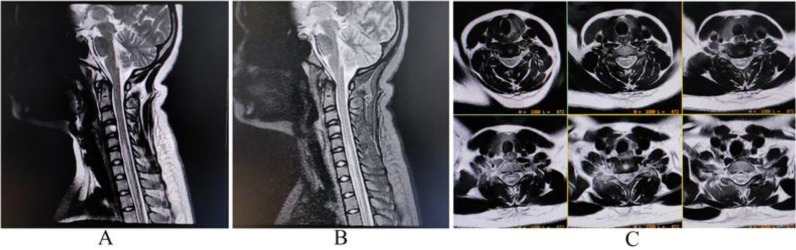
Magnetic resonance imaging of the spinal cord revealed a lesion showing a hyperintense signal on sagittal tbl2-weighted imaging (A), sagittal short tau inversion recovery sequence (B), and axial tbl2-weighted imaging (C), indicating a longitudinally extensive demyelinating lesion from the C5 to the D4 level.

